# Supplementary material for: Quadrupole topological photonic crystals
Source: Nat Commun. 2020 Jun 19;11:3119. doi: 10.1038/s41467-020-16916-z (PMC7305167; doi:10.1038/s41467-020-16916-z)
Supplement: Supplementary file 1 — Supplementary information [file 41467_2020_16916_MOESM1_ESM.pdf]

Supplementary Information - Quadrupole Topological Photonic Crystals

He et al.

Supplementary Note 1: Calculating Wannier bands of photonic crystals

Our calculations of (Wannier) band polarizations using (nested) Wilson loop follow that of the paper by Benalcazar *et al*[1]. To precede, we first define the Wilson line along x direction as

$$F_{x,\mathbf{k}}^{mn} = \langle u_{\mathbf{k}+\Delta_x}^m | \, \varepsilon \, | u_{\mathbf{k}}^n \rangle = \int_{\text{unit cell}} dxdy \; u_{\mathbf{k}+\Delta_x}^{m*}(x,y)\varepsilon(x,y)u_{\mathbf{k}}^n(x,y) \tag{1}$$

where $|u_{\mathbf{k}}\rangle$ is the cell-periodic electric field (E_z for TM mode) satisfies the orthonomal condition $\langle u_{\mathbf{k}}^m | \, \varepsilon \, | u_{\mathbf{k}}^n \rangle = \delta_{mn}$. $\varepsilon(x,y)$ is the dielectric permittivity. $\Delta_x = \frac{2\pi}{aN_x}\hat{\mathbf{x}}$ (we set lattice constant $a = 1$ hereafter) and N_x is the number of unit cells along x direction. m, $n \in 1 \ldots N_{occ}$ denote band indices below the energy gap of interest. Accordingly, the Wilson loop along x direction is defined as

$$W_{x,\mathbf{k}_i+2\pi\hat{\mathbf{x}}\leftarrow\mathbf{k}_i} = F_{x,\mathbf{k}_i+2\pi\hat{\mathbf{x}}-\Delta_x} \cdots F_{x,\mathbf{k}_i+\Delta_x} F_{x,\mathbf{k}_i} \tag{2}$$

The Wannier centers $\{\nu_x^j(k_y)\}$ are encoded in the phases of the Wilson loop eigenvalues

$$W_{x,\mathbf{k}} \, |v_{x,\mathbf{k}}^j\rangle = e^{i2\pi\nu_x^j(k_y)} \, |v_{x,\mathbf{k}}^j\rangle \tag{3}$$

where

$$F_{x,\mathbf{k}} \, |v_{x,\mathbf{k}}^j\rangle = e^{i\Delta_x\nu_x^j(k_y)} \, |v_{x,\mathbf{k}+\Delta_x}^j\rangle \tag{4}$$

The polarization reads

$$\nu_x(k_y) = \sum_j^{N_{occ}} \nu_x^j(k_y) = \frac{-i}{2\pi} \ln \det W_{x,\mathbf{k}} \tag{5}$$

The corresponding Wannier function that is localized around R_x and extended along y is

$$\begin{aligned} |\Psi_{R_x,k_y}^j\rangle &= \frac{1}{\sqrt{N_x N_y}} \sum_{n=1}^{N_{occ}} \sum_{k_x} e^{-ik_x R_x} e^{i\mathbf{k}\cdot\mathbf{r}} [v_{x,\mathbf{k}}^j]^n \, |u_{\mathbf{k}}^n\rangle \\ &= \frac{1}{\sqrt{N_x N_y}} \sum_{k_x} e^{-ik_x R_x} e^{i\mathbf{k}\cdot\mathbf{r}} \, |w_{x,\mathbf{k}}^j\rangle \end{aligned}$$

where we define the Wannier band basis as

$$|w_{x,\mathbf{k}}^{j}\rangle = \sum_{n=1}^{N_{occ}} [v_{x,\mathbf{k}}^{j}]^{n} |u_{\mathbf{k}}^{n}\rangle \tag{6}$$

Similarly, the nested Wilson line along y direction for a Wannier sector ν_x is defined as

$$\tilde{F}_{y,\mathbf{k}}^{\nu_x} = \langle w_{x,\mathbf{k}+\Delta_y}^{j}| \varepsilon |w_{x,\mathbf{k}}^{j'}\rangle \tag{7}$$

where $j, j' \in 1 \dots N_W$ are all Wannier bands within the Wannier sector ν_x. The nested Wilson loop is

$$\tilde{W}_y = \tilde{F}_{y,\mathbf{k}_i+2\pi\hat{\mathbf{y}}-\Delta_y} \cdots \tilde{F}_{y,\mathbf{k}_i+\Delta_y} \tilde{F}_{y,\mathbf{k}_i} \tag{8}$$

The polarizations of the Wannier bands are encoded in the phases of the nested Wilson loop eigenvalues

$$\tilde{W}_y |v_{y,\mathbf{k}}^{\nu_x,j}\rangle = e^{i2\pi p_y^{\nu_x,j}(k_x)} |v_{y,\mathbf{k}}^{\nu_x,j}\rangle \tag{9}$$

and the nested Wannier band polarization reads

$$p_y^{\nu_x}(k_x) = \sum_{j}^{N_W} p_y^{\nu_x,j}(k_x) = \frac{-i}{2\pi} \ln \det \tilde{W}_{y,\mathbf{k}} \tag{10}$$

Supplementary Note 2: Symmetry constraints on Wannier bands and topological invariants

Symmetries impose certain constraints on the (nested) Wannier band polarizations:

$$\{\nu_x^j(k_y)\} \overset{C_2}{=} \{-\nu_x^j(-k_y)\} \quad \mathrm{mod}\ 1 \tag{11a}$$

$$\{\nu_x^j(k_y)\} \overset{C_4}{=} \{\nu_y^j(-k_x)\} \quad \mathrm{mod}\ 1 \tag{11b}$$

$$p_x^{\nu_y^-}(k_y) \overset{M_xT}{=} -p_x^{\nu_y^-}(-k_y) \quad \mathrm{mod}\ 1 \tag{11c}$$

$$p_y^{\nu_x^-}(k_x) \overset{M_yT}{=} -p_y^{\nu_y^-}(-k_x) \mod 1 \tag{11d}$$

These relations can be proved by analyzing the transformation properties of the (nested) Wilson loop under symmetry operations[1]. Therefore, for the PhCs presented in the main text, the two Wannier bands $\{\nu_x^j(k_y)\}$ are odd with respect to the origin. The Wannier bands $\{\nu_x^j(k_y)\}$ and $\{\nu_y^j(k_x)\}$ are also related by C_4 symmetry. Meanwhile, the presence of M_xT and M_yT quantize the polarization of each Wannier sector, namely, $p_x^{\nu_y^-}$, $p_y^{\nu_x^-}$ = 0 or 1/2. For a quadrupole topological insulator considered in the main text, we have $p_x^{\nu_y^\pm} = p_y^{\nu_x^\pm} = 1/2$.

Supplementary Note 3: Calculating the expectation value of the quadrupole operator in a PhC

Consider a 2D system of length L_x and L_y that has periodic boundary conditions in both directions. The polarization p_x can be calculated as an expectation value of the operator $\exp\left(i\frac{2\pi x}{L_x}\right)$ proposed by Resta[2]. Specifically, the polarization can be easily evaluated as

$$p_x = \frac{1}{2\pi}\mathrm{Im}\ln\det S \tag{12}$$

where

$$S_{\mathbf{k}n,\mathbf{k}'n'} = \int_0^{L_x}\int_0^{L_y} dxdy\ \psi^*_{\mathbf{k},n}(x,y)\ \exp\left(i\frac{2\pi x}{L_x}\right)\varepsilon(x,y)\ \psi_{\mathbf{k}',n'}(x,y) \tag{13}$$

where $\psi_{\mathbf{k}n}$ is the Bloch wave function with wave vector $\mathbf{k}$ and n denotes band index. It can be easily shown that the above equation is equivalent to the integral of the trace of the non-Abelian Berry connection $A_{\mathbf{k}}^{nn'}$ across the Brillouin zone.

As suggested recently by Wheeler *et al*[3], the bulk quadrupole moment q_{xy} can also be calcu-

lated as the expectation value of the quadrupole operator $\exp\left(i\frac{2\pi xy}{L_x L_y}\right)$ in a similar fashion

$$q_{xy} = \frac{1}{2\pi}\mathbf{Im}\ln\det S \tag{14}$$

where

$$S_{\mathbf{k}n,\mathbf{k}'n'} = \int_0^{L_x}\int_0^{L_y} dxdy\ \psi^*_{\mathbf{k},n}(x,y)\ \exp\left(i\frac{2\pi xy}{L_x L_y}\right)\varepsilon(x,y)\ \psi_{\mathbf{k}',n'}(x,y) \tag{15}$$

Our numerical calculation shows $q_{xy} = 1/2$ and 0 for quadrupole and trivial insulators, respectively, consistent with the conclusions obtained from symmetry analysis and nested Wilson loop calculations presented in the main text.

Supplementary Note 4: Quadrupole topological photonic crystals with M_xT and M_yT symmetries

The bulk quadrupole moment can be quantized either by four-fold rotation symmetry or the product of mirror and time-reversal symmetries. In the following, we analyze the quadrupole topological order for PhCs with M_xT and M_yT by calculating the nested Wilson loop. To keep M_xT and M_yT symmetries and break C_4 symmetry, we displace the four rods within a unit cell as shown in Supplementary Figure 1a. Depending on the sign of d_x and d_y (here d_x and d_y are defined as the relative displacements of the right-top rod with respect to the supercell structure), we find the resulting photonic crystals are either quadrupole insulators or trivial insulators, all with quantized Wannier band polarizations, as required by Supplementary Equation 11c,d. Supplementary Figure 1b shows the complete phase diagram as a function of d_x, d_y, which features four regions with $(p_x^{\nu_y^-}, p_y^{\nu_x^-}) = (0,0), (0,1/2), (1/2,0), (1/2,1/2)$, being separated by gapless phases (red lines). The representative nested Wannier bands for the four phases are shown in Supplementary Figure

2. In particular, the bulk quadrupole moment $q_{xy} = 2p_x^{\nu_y^-} p_y^{\nu_x^-} = 1/2$ only when d_x and d_y are both positive (Supplementary Figure 2d), whereas other displacements lead to $q_{xy} = 0$. This calculation is consistent with the bulk quadrupole moment evaluated using the quadrupole operator.

Supplementary Note 5: Intuitive understanding of the difference between trivial and non-trivial PhCs

The quadrupole and trivial PhCs in Fig. 2 in the main text are related by shifting the center of the unit cell by half a period $(1/2, 1/2)$. Specifically, the Wannier band polarizations will change by $1/2$ in accordance with the coordinate shift, as can be inferred from Supplementary Figure 1b. The resulted quadrupole moment is

$$\tilde{q}_{xy} = 2(p_x^{\nu_y^-} + 1/2)(p_y^{\nu_x^-} + 1/2) = q_{xy} + p_x^{\nu_y^-} + p_y^{\nu_x^-} + 1/2 \tag{16}$$

For the trivial insulator presented in the main text, we have $q_{xy} = p_x^{\nu_y^-} = p_y^{\nu_x^-} = 0$. Therefore, the structure after coordinate shift is topologically non-trivial with $\tilde{q}_{xy} = 1/2$.

Supplementary Note 6: Calculating spatially resolved polarization density

Consider a semi-infinite strip with periodic boundary condition along x and closed boundary condition along y. According to the preceding discussion, the Wannier functions take the form

$$|\Psi_{R_x}^j\rangle = \frac{1}{\sqrt{N_x}} \sum_{n=1}^{N_{occ}\times N_y} \sum_{k_x} [v_{k_x}^j]^n e^{-ik_x R_x} e^{ik_x x} |u_{n,k_x}\rangle \tag{17}$$

where N_y is the number of unit cells along the y direction and $j \in 1 \dots N_{occ} \times N_y$. The density of

the Wannier function with $R_x = 0$, $|\Psi_0^j\rangle$, along the y direction is

$$\rho(y)^j = \int_{-\infty}^{\infty} dx \int_{y}^{y+a} dy \; \Psi_0^{j*}(x,y) \; \varepsilon(x,y) \; \Psi_0^j(x,y) \tag{18}$$

Therefore, the spatially resolved polarization density is

$$p_x(y) = \sum_{j=1}^{N_{occ} \times N_y} \nu_x^j \rho(y)^j \tag{19}$$

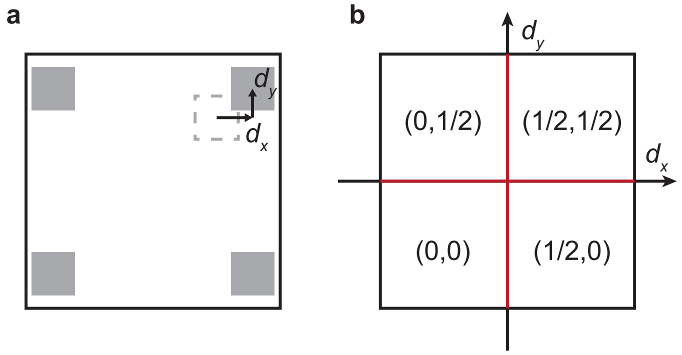

Supplementary Figure 1: | **Topological phase diagram of photonic crystals with mirror symmetries. a,** Unit cell of the perturbed PhCs with M_xT and M_yT symmetries. The right-top rod is displaced by (d_x, d_y) with respect to the supercell structure. The structure dose not have C_4 symmetry when $d_x \neq d_y$ **b,** Displacements with positive d_x and d_y give rise to quadrupole phases with $(p_x^{\nu_y^-}, p_y^{\nu_x^-})$ = (1/2, 1/2). In all other cases, the displacements lead to trivial phases.

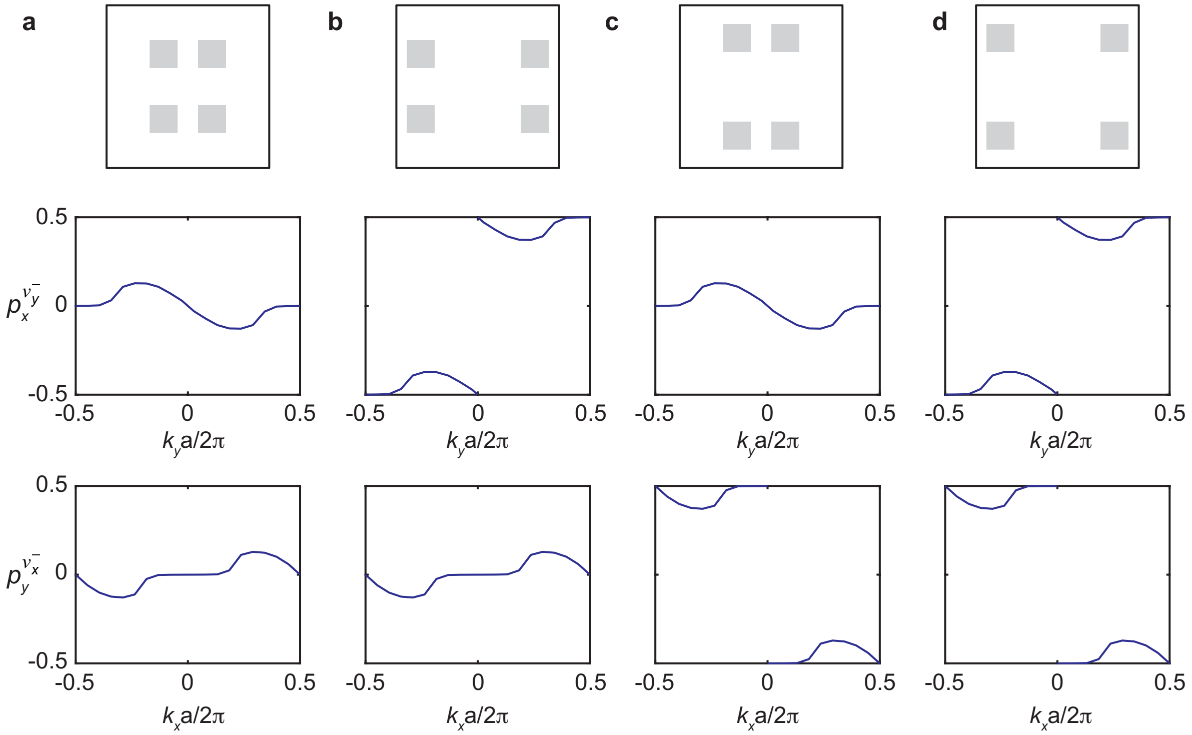

Supplementary Figure 2: | **Nested Wannier bands for PhCs with mirror symmetries. a-d,** Nested Wannier bands polarizations $p_x^{\nu_y^-}(k_y)$ and $p_y^{\nu_x^-}(k_x)$ are calculated for displaced PhCs. Results show $p_x^{\nu_y^-}$ is quantized to 0.5 (0) for positive (negative) displacement d_x. Similar conclusion applies to $p_y^{\nu_x^-}$.

Supplementary References

1. Benalcazar, W. A., Bernevig, B. A. & Hughes, T. L. Electric multipole moments, topological multipole moment pumping, and chiral hinge states in crystalline insulators. *Physical Review B* **96**, 245115 (2017).

2. Resta, R. Quantum-mechanical position operator in extended systems. *Physical Review Letters* **80**, 1800 (1998).

3. Wheeler, W. A., Wagner, L. K. & Hughes, T. L. Many-body electric multipole operators in extended systems. *Physical Review B* **100**, 245135 (2019).
